# Automatic Classification of Online Discussions and Other Learning Traces to Detect Cognitive Presence

**DOI:** 10.1007/s40593-023-00335-4

**Published:** 2023-05-01

**Authors:** Verena Dornauer, Michael Netzer, Éva Kaczkó, Lisa-Maria Norz, Elske Ammenwerth

**Affiliations:** 1 Institute of Medical Informatics, UMIT TIROL – Private University for Health Sciences and Health Technology, Eduard Wallnöfer Zentrum 1, Hall in Tirol, 6060 Austria; 2 Department of Organisation and Learning, University of Innsbruck, Innsbruck, 6020 Austria

**Keywords:** Cognitive presence, Community of Inquiry, Machine learning, Classification

## Abstract

Cognitive presence is a core construct of the Community of Inquiry (CoI) framework. It is considered crucial for deep and meaningful online-based learning. CoI-based real-time dashboards visualizing students’ cognitive presence may help instructors to monitor and support students’ learning progress. Such real-time classifiers are often based on the linguistic analysis of the content of posts made by students. It is unclear whether these classifiers could be improved by considering other learning traces, such as files attached to students’ posts. We aimed to develop a German-language cognitive presence classifier that includes linguistic analysis using the Linguistic Inquiry and Word Count (LIWC) tool and other learning traces based on 1,521 manually coded meaningful units from an online-based university course. As learning traces, we included not only the linguistic features from the LIWC tool, but also features such as attaching files to a post, tagging, or using terms from the course glossary. We used the *k*-nearest neighbor method, a random forest model, and a multilayer perceptron as classifiers. The results showed an accuracy of up to 82% and a Cohen’s κ of 0.76 for the cognitive presence classifier for German posts. Including learning traces did not improve the predictive ability. In conclusion, we developed an automatic classifier for German-language courses based on a linguistic analysis of students’ posts. This classifier is a step toward a teacher dashboard. Our work also provides the first fully CoI-coded German dataset for future research on cognitive presence.

## Introduction

Online-based learning has been on the rise for many years. Several frameworks exist that help teachers build sustainable and well-suited learning experiences for their students. Online learning implies that the students are physically distant from the teacher and use technology (e.g., a computer) to access learning material and engage in discussions with the teacher or other students (Ally, 2004). One specific form of online-based learning is collaborative online learning, where students learn asynchronously in a group and are in contact with each other through asynchronous forum discussions (Garrison et al., 2000).

A well-known framework for collaborative learning is the Community of Inquiry framework, which recently celebrated 20 years of existence (Castellanos-Reyes, 2020). The Community of Inquiry framework describes how to achieve meaningful educational experiences within a group of students in a collaborative learning environment (Garrison et al., 2000).

The Community of Inquiry framework highlights three presences that are essential for a sustainable and well-suited educational experience: cognitive presence, social presence, and teaching presence. Cognitive presence, the presence this study deals with, defines the extent to which students can construct and confirm meaning through sustained reflection and discourse in a critical community of inquiry (Garrison et al., 2000). Cognitive presence is associated with high-order and critical thinking (Garrison et al., 2001). Critical thinking is generally one major education goal of universities for their students. Cognitive presence comprises four phases a student goes through in the critical thinking process (Garrison et al., 2001): triggering event, exploration, integration, and resolution. Higher levels of cognitive presence are associated with learning outcomes as indicated by course grades (Lee et al., 2022).

Social presence, by definition, is the ability of students in a community of inquiry to project themselves socially and emotionally as “real people” through emotional expression, open communication, and activities of group cohesion (Garrison et al., 2000). Social presence is essential to build a community of inquiry (Garrison et al., 2000).

Teaching presence is critical in fostering cognitive presence (Garrison, 2017; Redstone et al., 2018; Stenbom, 2018). For example, to foster cognitive presence, the teacher provides multiple opportunities for students to engage with each other and with the course content (Moore & Miller, 2022). The nature of the defined task (e.g., case-based discussion) and the facilitation of students’ discussions impact cognitive presence (Darabi et al., 2011; Sadaf & Olesova, 2017). Specific instructional techniques (question prompts by the teacher) and guidelines (such as post deadlines and a minimum number of responses) help engage learners in online dialogue (Moore & Miller, 2022).

To decide on appropriate instructional techniques to foster cognitive presence, the teacher needs information on the level of cognitive presence. This usually happens by reading all the students’ posts. For example, the teacher may follow all discussion threads to see whether students are still in the exploration phase or if they are already heading toward integration or resolution. For this, the teacher may analyze the posts’ content, wording, or timing.

Reading all discussion posts, however, is time-consuming and may not be feasible in a larger course. Instead, real-time automatic analysis of online discussions could help the teacher to determine the students’ cognitive learning process efficiently and to act accordingly. Automatic analysis of the cognitive presence phases would thus help teachers diagnose how students are doing in the expected learning process. For example, suppose the task is designed to reach the resolution phase, but the automatic analysis shows that students continue to explore ideas. In that case, the teacher could intervene and ask questions to help them move on to the integration phase (e.g., questions to help connect ideas, justify hypotheses, or create solutions). It would also be feasible for the teacher to pre-define questions for each phase. These questions would be automatically posted in the learning management system depending on the level of cognitive presence reached. The automatic analysis could even help students reflect on where they are in their learning process. Especially in MOOCs with a high number of students’ posts, such automatic analysis of the level of cognitive presence could be beneficial since it would be impossible for the teacher to read all the posts and respond accordingly.

The idea of analyzing data on the students’ learning process and presenting this data to the teacher (or students) is not new. The tool for this has been termed a learning analytics dashboard (Klerkx et al., 2017). Teacher-faced dashboards are tools to capture and visualize, in aggregated and real-time form, information essential to make informed decisions about students’ learning activities. Teacher dashboards have the “potential of making the ‘invisible’ visible to teachers” and so may enhance “their ability to engage students more effectively” (Comber et al., 2018). Learning management systems typically provide a basic teacher dashboard offering essential information such as students’ online time and activities, number and content of written posts, and assessment results. To our knowledge, real-time analysis of the level of cognitive presence of students is not yet available. This analysis would give teachers additional information, allowing them to better support their students’ learning processes.

The precondition for real-time analysis of cognitive presence is the automated measurement of cognitive presence within the student group. Automatic measurement analyzes the content of students’ posts and tries to classify these automatically into the four phases of cognitive presence (triggering, exploration, integration, and resolution). Automatic measurement for cognitive presence may be added to a teacher dashboard to monitor students’ cognitive engagement in a course (Lee et al., 2022). Such a teacher dashboard could be part of the learning management system and describe cognitive presence both on the level of an individual student and on the level of the whole group. The teacher could then see the cognitive presence and how the group develops over time, e.g., from lower levels of cognitive presence at the beginning to higher levels of cognitive presence toward the end of the course, and intervene as described above.

For such an automatic measurement, automatic classifiers need to be developed to analyze the students’ posts and classify the post into the four levels of cognitive presence. Automatic classifiers for cognitive presence have already been developed and tested for the English and Portuguese languages (Barbosa et al., 2021; Kovanović et al., 2016; Neto et al., 2018). A classifier for German-language posts is still lacking. A language’s specific structural and linguistic features may influence the prediction of the cognitive presence phase. It thus seems essential to assess whether these earlier classifiers are also sufficiently accurate for German posts.

Besides analyzing the content of students’ posts, as earlier authors have done, our idea is also to exploit so-called learning traces. We define a learning trace as something other than the pure text content of a student’s post that may indicate cognitive presence. For example, cognitive presence may also be visible in whether students attach a solution to a post, structure it through formatting, embed a picture to it, or cite literature.

In the following sections, we will provide a short overview of the Community of Inquiry framework, primarily of the cognitive presence construct, and of research concerning the automated classification of cognitive presence. We then describe our methodological procedure and our data sources. We show the results using four variations of the automated classifier and provide insights into the features we used. In the end, we will discuss the lessons learned and the next steps toward a CoI teacher dashboard.

### The Community of Inquiry Framework and Cognitive Presence

Cognitive presence was developed by Garrison et al. (2001) based on Dewey’s Practical Inquiry Model (Dewey, 1933). The Practical Inquiry Model illustrates the process of critical thinking in the following four phases (Garrison et al., 2001):

#### Triggering Event

The students’ interest is aroused, and the problem to be solved is identified and recognized by the students. Triggering starts mainly with the teacher, but students can also trigger other students.

#### Exploration

The students explore the problem to be solved through individual research for information and reflection on that information, alternating with phases of communication in the learning community.

#### Integration

The knowledge gained is integrated and considered to solve the problem and create solutions.

#### Resolution

The solution is tested by real-life application through thought experiments and is defended or justified.

The occurrence of these phases is not always sequential. Students may move back and forth through the phases (Garrison, 2017).

### Automated Classifiers for Cognitive Presence

Earlier research has developed classifiers for cognitive presence in English and Portuguese (Table 1).

**Table 1:** Automated classifiers for cognitive presence

Authors	Machine learning algorithm	Language of students’ posts	Computer–human interrater reliability (Cohen’s *κ*, lowest–highest score)
(McKlin et al., 2001)	Neural Network	English	*κ* = 0.31–0.76
(McKlin, 2004)	Neural Network	English	*κ* = 0.52–0.70
(Corich et al., 2006)	Bayesian Network	English	*κ* = 0.65–0.71
(Kovanović et al., 2014)	Support Vector Machine	English	*κ* = 0.41
(Waters et al., 2015)	Conditional Random Fields	English	*κ* = 0.48
(Kovanović et al., 2016)	Random Forest	English	*κ* = 0.63
(Farrow et al., 2019)	Random Forest	English	*κ =* 0.38–0.63
(Hayati et al., 2019)	Naive Bayes Classifier Support Vector Machine Logistic Regression	English	*κ* = 0.69 (NBC) *κ* = 0.61 (SVM) *κ* = 0.53 (LR)
(Neto et al., 2018)	Random Forest	Portuguese	*κ* = 0.72
(Barbosa et al., 2020)	Random Forest	Cross-Language (Portuguese on English classifier)	*κ* = 0.32
(Barbosa et al., 2021)	Random Forest	i) original English (E) ii) translation from English to Portuguese (E–P) iii) original Portuguese (P) iv) translation from Portuguese to English (P–E)	*i) κ* = 0.38 (E) *ii) κ* = 0.38 (E–P) *iii) κ* = 0.52 (P) *iv) κ* = 0.69 (P–E)
(Neto et al., 2021)	Random Forest	English	*κ =* 0.49–0.62
(Hu et al., 2022)	Random Forest	English	*κ =* 0.54

Research initially started with methods of deep learning (McKlin et al., 2001) and then assessed the support vector machine classifier (Hayati et al., 2019; Kovanović et al., 2014), conditional random fields (Waters et al., 2015), naive Bayes classifier, and logistic regression (Hayati et al., 2019). Recently, research has focused on random forest classifiers in different variations (Barbosa et al., 2020, 2021; Farrow et al., 2019; Hu et al., 2022; Kovanović et al., 2016; Neto et al., 2018, 2021).

All authors used Cohen’s *κ* to measure computer–human interrater reliability (Cohen, 1960). The highest Cohen’s *κ* scores were achieved in the English language by McKlin et al. using methods of deep learning (0.76) (McKlin et al., 2001) and in the Portuguese language by Neto et al. using a random forest classifier (0.72) (Neto et al., 2018).

Several studies developed their classifiers based on the same dataset, i.e., using the same set of students’ posts from the same course. For example, several studies used a dataset from only one postgraduate software engineering online course (Barbosa et al., 2020, 2021; Farrow et al., 2019; Kovanović et al., 2014; Waters et al., 2015). Only Hu et al. applied their classifier on a philosophy dataset and validated their classifier on three disciplines (medicine, education, and humanities) (Hu et al., 2022). Further, Neto et al. applied a random forest classifier to a biology and technology dataset (Neto et al., 2018, 2021).

Frequently, results show an imbalance in the occurrences of the phases of cognitive presence. This problem has frequently been addressed by applying the oversampling strategy SMOTE (Synthetic Minority Oversampling Technique), as initially used by Kovanović et al. (2016) and demonstrated in detail by Farrow et al. (2019). The studies for the Portuguese language also employed SMOTE (Neto et al., 2018), with one study using a specific pipeline with the steps NearMiss, Tomek, SMOTE + Tomek, and Edited Nearest Neighbor (Barbosa et al., 2020).

Earlier research has used four feature types to classify student posts into the phases of cognitive presence: textual features, structural features, LIWC features, and Coh-Metrix features. Textual features can be defined as features based directly on the raw student posts. For example, n-grams are groups of words considered a unit for the analysis (Kowsari et al., 2019). Another example is the application of doc2vec, which transforms text into numerical vectors as used by Hayati et al. (2019). Other authors who have applied textual features are McKlin et al. (2001), Corich et al. (2006), Kovanović et al. (2014), and Waters et al. (2015).

Structural features, as used by Kovanović et al. (2014), Waters et al. (2015), Hu et al. (2022), and Neto et al. (2021) describe the location of posts in a sequence of discussions. Location means, for example, whether the post is an opening message or a reply or in which position it stands in a sequence of discussion posts.

LIWC features are numeric values calculated from the Language Inquiry and Word Count (LIWC) tool. The LIWC tool is widely used in the field of the social sciences (Kowsari et al., 2019). It classifies words into different psychological categories as cognitive processes (e.g., insight, causation, discrepancy), affective processes (e.g., positive and negative emotions), or social processes (e.g., female references, male references) (Meier et al., 2018; Tausczik & Pennebaker, 2010). Since 2016, starting with Kovanović et al. (2016), all authors have used these LIWC features in their classifiers of cognitive presence (Barbosa et al., 2020, 2021; Farrow et al., 2019; Hayati et al., 2019; Hu et al., 2022; Neto et al., 2018, 2021). The most predictive LIWC features in the English language based on students’ written artifacts were the number of question marks, the number of money-related words, and the number of first-person singular pronouns (Kovanović et al., 2016). The most predictive LIWC features in the Portuguese language were the number of question marks, the number of words larger than six letters, the number of articles, the number of prepositions, the number of conjunctions, the number of verbs, the number of quantifiers, and the number of pronouns in the third person singular (Neto et al., 2018).

Coh-Metrix features are numeric values that are calculated from the Coh-Metrix tool. The Coh-Metrix tool evaluates cohesion, language, and readability (Graesser et al., 2004). All mentioned authors used these Coh-Metrix features in their classifiers of cognitive presence (Barbosa et al., 2020, 2021; Farrow et al., 2019; Hu et al., 2022; Kovanović et al., 2016; Neto et al., 2018, 2021). The Coh-Metrix tool is, according to its documentation, only available for English-language texts. To our knowledge, the only other language besides English that it has also been implemented in is Portuguese (Neto et al., 2018).

No research to our knowledge has tried to exploit other learning traces (such as adding attachments, embedding figures or videos in the posts, or citing literature) as indicators of integrating new knowledge or showing advanced understanding and thus of cognitive presence. The published classifiers for cognitive presence, until now, have not included this information to optimize the accuracy of the classifier.

A classifier trained in English can only classify posts in the English language. Barbosa et al. attempted a cross-language approach where the Portuguese language was classified by the classifier trained in English, resulting in a Cohen’s *κ* of 0.32 (Barbosa et al., 2020). An alternative line of research has translated students’ posts from Portuguese into English and vice versa and then applied the English classifier; the authors achieved a Cohen’s *κ* of 0.69 (Barbosa et al., 2021). For comparison, Cohen’s *κ* of Portuguese text and classification by a Portuguese-trained classifier stood at 0.72 (Neto et al., 2018).

To summarize, earlier research developed classifiers for cognitive presence for posts in English and Portuguese. A German-language classifier would be needed to pursue our vision of real-time monitoring in a teacher dashboard in German-speaking countries. In addition, available classifiers focused on the textual content of students’ posts and did not exploit information on other learning traces.

Our research question was thus: Which predictive power can we reach for an automatic classifier for German-language posts based on linguistic features and learning traces?

## Experimental Setup and Methods

### Dataset

We used data from an online-based course in Software Quality Engineering that was part of a postgraduate master’s program in Health Information Management at the UMIT TIROL university in Austria. Fifteen students attended the course. All students were adults and working part or full-time. The mean age was 41 years (range 28–49). Half of the students lived in Germany; the others came from Austria, Switzerland, and France. The students had professional backgrounds in nursing, medical information management, medical informatics or computer science, medicine, or pharmacy.

In total, 1,147 student posts from all 15 students were considered in this study. The number of posts ranged from 36 (minimum) to 102 (maximum) per student, with a median of 64. On average, students used 175 words per post. The language of the course was German. In the course, teachers gave weekly work assignments. The students had to complete each work assignment and discuss their solutions with their peers. The teacher served as a learning coach who guided the student group through the course. Communication took place in asynchronous forum discussions. At the end of the course, students had to complete a final graded assignment. Student participation in the asynchronous forum discussions influenced the final grade. The university’s ethics board evaluated and approved the whole study process. Student posts were immediately anonymized following export from the in-house learning management system.

### Quantitative Content Analysis – Preparation of The Dataset

First, we manually classified all students’ posts to the different phases of cognitive presence. The coding categories for cognitive presence (triggering event, exploration, integration, resolution) were based on the coding scheme for cognitive presence described by Garrison et al. (2001), which we translated into German and adapted to the course setting.

Students’ posts may comprise a discussion addressing more than one phase of cognitive presence. A post may, for example, comprise an exploration part and an integration part (see Table 2). In those cases where we found that a post addressed more than one phase, we split the post into two parts (and, in the rare cases of quite long posts, into three or more parts). We named these parts “meaningful units”. Each meaningful unit was then separately coded to a phase of cognitive presence. The two coders defined these meaningful units together and reached a consensus. We assumed that splitting posts into meaningful units would result in a higher precision of our automatic classifier.

**Table 2:** Coding example with two meaningful units (translated from German to English)

Original post	Coded phases of cognitive presence
“Re: My IT project – dunning system Hi < name>, interesting to read how this all looks from a scrum master’s perspective. We definitely proceeded in our project as described above. For me, it really looks like scrum, because here we finished part by part. But we knew from the beginning what we wanted. Theoretically, it would have been possible to express wishes during the creation of the dunning system and to commission them during the production process. Since the dunning project is very small and manageable, it was no rocket science for us to figure out what we needed from start to finish. In addition, we have already commissioned add-ins of this kind on several occasions. It may be due to our experience that we did not have to proceed incrementally. To return to scrum, the dunning system is a small part of a large system with many functions. I think building and expanding the system is a real (giant) scrum project. Would you have used another name for the creation cycle of the dunning system? Best regards to Berlin < name>” *(message #132, translated from German)*	Resolution phase because the student defends the solution: “We definitely proceeded […] we did not have to proceed incrementally.” Exploration phase because of suggestion for consideration: “To come back to scrum […] of the dunning system?”

In addition, we coded the presence of learning traces in the posts (see Table 3). These learning traces were coded as “1” if present. The learning traces were directly identified in the learning management system and cross-checked between the two coders.

**Table 3:** Coded learning traces with an explanation

Coded learning traces	Explanation
Glossary link	Students use words from the course glossary (students use course terminology). The teacher prepared this course glossary in the learning management system in advance of the course. When students use a term from the glossary (e.g., “health information system”), the learning management system automatically links this term to the glossary if used in the students’ posts. Students could thus quickly look up any unclear terminology. We assumed that using glossary terms could indicate a higher level of cognitive presence.
Literature	Students cite literature. We assumed that searching for and including references could indicate a higher level of cognitive presence.
Pre-knowledge	Students describe their preliminary knowledge of the topic of the module. This prior knowledge depends on the professional background of the students. In the first week of the course, students were asked to describe whether they had any earlier experience with the course topic. Students can integrate pre-knowledge into the course. We assumed that this could allow them to reach a higher level of cognitive presence.
PDF solution:	Students attach a file with a solution to the post. We assumed this could indicate a higher level of cognitive presence as the students invested more time developing and presenting their solutions.
Other PDF	Students attach a file with content other than a solution. We assumed this could indicate a higher level of cognitive presence as the students spent time finding and aggregating relevant content.
Picture	Students embed a picture within the post. We assumed this could indicate a higher level of cognitive presence as the students spent time searching for or developing a visual representation of information or results.
Structuring	Students structure the post, e.g., by formatting it into sections. We assumed that this could indicate a higher level of cognitive presence as it may represent a better-structured internal knowledge on the part of the students.
Tags	Students assign tags to the post. We assumed that this could indicate a higher level of cognitive presence as the students spent time classifying and summarizing the content of a discussion.
URL	Students embed a URL in the post. We assumed this could indicate a higher level of cognitive presence as the students searched for and integrated additional information into their argumentation.
Video	Students embed a video related to the course topics within the post. We assumed that this could indicate a higher level of cognitive presence as the students integrated visual material into their argumentation.

Two coders independently conducted the manual quantitative content analysis on all posts using MAXQDA (MAXQDA Plus 2020 Release 20.3.0) based on our German CoI codebook. MAXQDA is a standard data analysis tool for qualitative data (in our case, the content of the students’ posts).

Coding was conducted using a structured approach, in line with earlier studies on automatic analysis of cognitive presence, such as Neto et al. (2018). First, two coders coded 50 posts together. Afterward, each coder coded the posts in blocks of 150 separately. After each block, the interrater agreement was assessed, and any differences in coding were resolved through discussion. In the beginning, the interrater agreement was low at 30%. After every 150 posts, the interrater agreement was higher than before. If no consensus could be reached between the two coders, a third person reviewed the discussion and decided. After this consensus process, the final interrater agreement for coding was excellent, with nearly 98%. Only in very few cases no consensus could be reached among the three researchers. This coding procedure allowed us to start the machine learning process based on an agreed coding.

After this manual quantitative content analysis, our dataset comprised 1,521 meaningful units (identified within 1,147 posts). Each meaningful unit was assigned to exploration, integration, or resolution. Also, information on learning traces was documented for each meaningful unit. A triggering event was only coded once within the quantitative content analysis, so we excluded this phase from further analyses.

### Machine Learning Pipeline and Feature Engineering – LIWC Features and Learning Traces

We performed all analyses in Python 3.7. The following Python packages were used: pandas and NumPy for data handling, scikit-learn for implementing the machine learning classifiers, imbalanced-learn for over- and undersampling of the dataset, and matplotlib and seaborn for data visualization.

We applied two kinds of features: LIWC features and learning traces. We chose LIWC as it has been used in all text classification machine learning models of cognitive presence in recent years and as it is also available in German. We added learning traces as they may represent non-textual features that help to classify cognitive presence more accurately. We did not add Coh-Metrix features as they are not yet available in German.

The LIWC features were generated with the German version of the Language Inquiry and Word Count (LIWC) tool (LIWC2015, Version 1.6.0, June 2019) (Meier et al., 2018). The LIWC tool counts words into different psychological categories (Tausczik & Pennebaker, 2010). Overall, the LIWC tool calculated the word count and 96 different language-specific and psychologically relevant meaning categories for each of the 1,521 meaningful units. We assumed all LIWC features to be potentially relevant for cognitive presence. In particular, we tested the following seven feature categories: (i) cognitive processes, (ii) insight, (iii) causation, (iv) discrepancy, (v) tentativeness, (vi) certainty, and (vii) differentiation. Finally, we performed analyses with all LIWC features.

The presence of the learning traces was one-hot encoded and added to the meaningful units. Using this one-hot encoding, each learning trace is represented by a specific feature with binary values representing its presence. For instance, if a glossary link and literature citation are present for a particular post, these two corresponding features have a value of “1” and the remaining features have a value of “0”. All features were standardly scaled to prevent the overinfluencing of the machine learning classifier by features with high quantitative expression.

### Independent and Dependent Variables for The Machine Learning Classifier

The independent variables of our machine learning were the features based on text and context factors of students’ posts (LIWC features, learning traces). The dependent variable was the cognitive presence phase (exploration, integration, resolution) or non-cognitive presence.

We used the following two datasets with different feature sets:

- Dataset 1 includes only LIWC features based on 1,521 meaningful units.
- Dataset 2 includes LIWC features and one-hot encoded learning traces based on 1,521 meaningful units.

### Learning Model, Sampling, and Validation

We applied a *k*-nearest neighbor classifier (KNN) grounded on the standard Euclidean metric and based on the following considerations: *k*-nearest neighbor classifiers are, except for the best-fit *k*-value and the distance function, non-parametric. They are well-suited to handle multi-class datasets, effective for text-based datasets, and generally easy to implement and interpret (Kowsari et al., 2019). To our knowledge, earlier authors have not yet tested *k*-nearest neighbor classifiers. A *k*-nearest neighbor algorithm classifies instances by considering the *k*-nearest neighbors based on the data classification of the training data depending on similarity (Boateng et al., 2020). One disadvantage is that *k*-nearest neighbor classifiers may become computationally expensive when datasets grow (“lazy learner”) (Kowsari et al., 2019).

We used a random forest classifier (RF) as a second approach. In principle, the random forest classifier is an ensemble of decision trees produced by bagging. This approach minimizes the learner’s variance, a common disadvantage of tree-based methods. One advantage of this approach is the possibility of calculating the feature’s importance. In particular, we calculated feature importance based on feature permutation, defined as the decrease in a model score when a single feature value is randomly shuffled (Breiman, 2001). This feature ranking approach has advantages compared to impurity-based methods concerning high-cardinality features.

As a third approach, we used a multilayer perceptron (MLP) classifier, namely a neural network (Taud & Mas, 2018).

We applied SMOTE (Synthetic Minority Oversampling Technique) with oversampling of all minority classes to the highest class based on five nearest neighbors to handle the imbalanced dataset. Finally, we applied SMOTEENN, which leads to denoising the dataset (Batista et al., 2004). SMOTEENN consists of SMOTE based on five nearest neighbors followed by ENN (Edited Nearest Neighbor), which removes examples, that would have been misclassified based on three nearest neighbors (Batista et al., 2004; Chawla et al., 2002).

We divided our dataset into a development set (75% of samples) and a holdout evaluation set (25% of samples). The development set was used for hyperparameter tuning using a 10-fold cross-validation strategy. We used a grid search to determine the hyperparameter of our classifiers based on accuracy. For the *k*-nearest neighbor classifier, we determined the best *k* for neighbors (min = 1, max = 20) and the weighting strategy (i.e., uniform or distance based). For the random forest classifier, we varied the parameters of maximum depth (3, 5, 10) and minimum samples per split (2, 5, 10). The search grid for the MLP includes different hidden layer sizes ([50,50,50], [50,100,50], [100]), activation functions (tanh and relu), solver (adam and sgd), and varying values for alpha (0.0001, 0.05) as well as the learning rate (constant, adaptive). The holdout evaluation set was then used to calculate accuracy, error rate, and F_1_ score.

We performed four variants of sampling scenarios.

- Scenario 1: Dataset 1 with standard scaling, SMOTE oversampling of minority classes to the number of the highest class.
- Scenario 2: Dataset 2 with standard scaling, SMOTE oversampling of minority classes to the number of the highest class.
- Scenario 3: Dataset 1 with standard scaling, SMOTEENN over- and undersampling to denoise the dataset.
- Scenario 4: Dataset 2 with standard scaling, SMOTEENN over- and undersampling to denoise the dataset.

## Results

### Quantitative Content Analysis

We manually identified and coded 1,522 meaningful units within 1,147 students’ posts. For the consecutive analysis, we excluded the triggering event phase, which we found in only one post. Consequently, a total of 1,521 meaningful units were considered and coded.

Table 4 shows the codes which were assigned to the meaningful units.

**Table 4:** Phase of Cognitive Presence in students’ posts (n = 1,522 meaningful units)

Phase	Coded meaningful units	Percentage
Exploration	727	48%
Integration	444	29%
Resolution	235	15%
Non-cognitive presence	115	8%
Total	1,521	100%

We now present the outcome of sampling scenarios 1–4 with performance scores. We compare the four scenarios using Cohen’s *κ*.

**Fig. 1:**
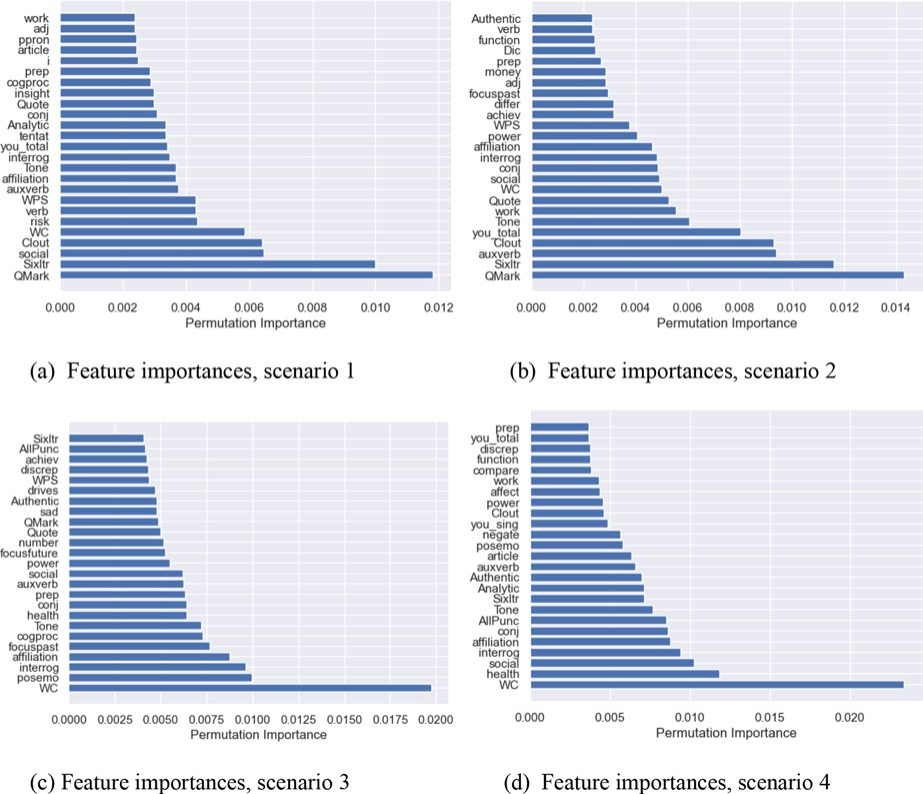
The top 25 features with the highest feature permutation importance based on the random forest classifier

Figure 1 presents the feature importance scores based on the random forest classifier for all four scenarios.

Features that are within the set of all four scenarios include “conj”, “interrog”, “Tone”, “affiliation”, “auxverb”, “WC”, “social”, and “Sixltr”. Table 5 explains these LIWC features in more detail.

**Table 5:** Description of the relevant LIWC features according to Meier et al. (Meier et al., 2018)

“conj”	Student uses conjunctions, e.g., instead, and, rather than
“interrog”	Student uses question words, e.g., when, why, how far
“tone”	Student shows verbal emotional tone
“affiliation”	Student uses words indicating affiliation, e.g., friend, social
“auxverb”	Student uses auxiliary verbs, e.g., (I) am or (I) have
“WC”	Word count
“social”	Student uses words that imply social activities, e.g., talk, buddy
“Sixltr”	Student uses words longer than 6 letters

The first variation was performed with Dataset 1, which comprised only standard-scaled LIWC features. We performed SMOTE oversampling. The highest accuracy on the holdout evaluation set for the first variation of the classification based only on LIWC features was reached by the MLP classifier at 0.82, with macro average values of 0.77 and a Cohen’s *κ* value of 0.76. The mean accuracy and the corresponding standard deviation on the development set were 0.81 ± 0.02.

The second variation was performed using Dataset 2, which comprised standard-scaled LIWC features and the other content learning traces. We performed SMOTE oversampling. The MLP classifier again obtained the highest performance on the evaluation set in terms of accuracy. Overall accuracy for the second variation of the classification based on LIWC features and other content learning traces stood at 0.82, with macro average values of 0.82 and a Cohen’s *κ* value of 0.76. The mean accuracy and the corresponding standard deviation on the development set were 0.80 ± 0.04.

The third variation was performed using Dataset 1, which comprised only standard-scaled LIWC features. We performed SMOTEENN over- and undersampling. The KNN and MLP classifier obtained the highest accuracy in this scenario. Both methods achieved an accuracy of 0.92. The corresponding Cohen’s *κ* values were 0.89 and 0.88. The corresponding standard deviation on the development set was 0.91 ± 0.02.

The fourth variation was performed using Dataset 2, which comprised standard-scaled LIWC features and the learning traces. We performed SMOTEENN over- and undersampling. The highest accuracy on the evaluation set was also obtained using an MLP classification approach. The corresponding accuracy was 0.91, with a macro average of 0.82 and a Cohen’s *κ* value of 0.87. The mean accuracy and the corresponding standard deviation on the development set were 0.91 ± 0.04.

Table 6 depicts the accuracy and Cohen’s *κ* value for each classifier and shows the F1 scores for the phases of exploration, integration, and resolution and for the non-cognitive presence (“other”) phase for all scenarios. The optimal parameter values for *alpha*, *hidden layer sizes*, *learning rate*, and *solver* of the multilayer perceptron classifier that were identified by the hyperparameter tuning step are identical for all simulations (*alpha = 0.05, hidden layer sizes = 100, learning rate = constant, solver = adam*). The optimal parameter *activation function* for scenario S1 was tangent hyperbolic (*tanh*) and rectified linear unit (*relu*) for scenarios S2, S3, and S4.

**Table 6:** Overall accuracies and corresponding Cohen’s κ values for all three considered scenarios using KNN, RF, and MLP classifier. The maximum values for each scenario (S) are highlighted in bold. F1 scores for exploration, integration, resolution, and non-cognitive presence phase and macro average value for all three considered scenarios using the classifier with the highest accuracy are shown in the lower part of the table. All performance values were calculated on an independent test set

Scenario	Model	Accuracy	Cohen’s κ	
**S1**	KNN	0.79	0.72	
	RF	0.77	0.70	
	**MLP**	**0.82**	**0.76**	
**S2**	KNN	0.77	0.69	
	RF	0.76	0.68	
	**MLP**	**0.82**	**0.76**	
**S3**	**KNN**	**0.92**	**0.89**	
	RF	0.87	0.80	
	MLP	0.92	0.88	
**S4**	KNN	0.89	0.83	
	RF	0.85	0.76	
	**MLP**	**0.91**	**0.87**	
	**F1 scores**			
**Phase**	**Scenario 1** **MLP**	**Scenario 2** **MLP**	**Scenario 3** **MLP**	**Scenario 4** **MLP**
**Exploration**	0.54	0.69	0.44	0.53
**Integration**	0.75	0.73	0.86	0.84
**Resolution**	0.83	0.85	0.93	0.91
**Non-cognitive presence**	0.98	0.99	0.99	1.00
**Macro average**	0.77	0.82	0.81	0.82

## Discussion

We aimed to implement and validate a German-language cognitive presence classifier that includes learning traces in addition to linguistic analysis of post content. We evaluated three different sampling scenarios using *k*-nearest neighbor, random forest, and neural network machine learning methods to classify the content of German-language student posts based on 1,521 meaningful units. In our dataset, the occurrence of integration and resolution was higher than in previous studies, e.g., Kovanović et al. (2016) and Neto et al. (2018, 2021). This could be because our work assignments were designed to urge students to reach integration and even resolution.

In sampling scenario 1, we deployed our machine learning classifier using the LIWC tool. Here, we achieved a “substantial” (Landis & Koch, 1977) agreement with a Cohen’s *κ* of 0.76.

In sampling scenario 2, we added learning traces to our machine learning classifier, e.g., the information that students formatted their posts, attached a document to their post, or embedded a video or a picture in their post. Interestingly, including these features did not improve the predictive ability. Our machine learning classifier also achieved “substantial” agreement here with a Cohen’s *κ* of 0.76 (Landis & Koch, 1977). The most essential learning traces were using a term from the course glossary, citing literature, describing pre-knowledge for the general topic of the course, attaching a file with a solution to the post, and formatting the post. For both analyses, we adjusted the number of meaningful units of students’ posts in the lower numerically occurring presence categories, following the example of earlier authors (Kovanović et al., 2016; Neto et al., 2018). The reason for doing this was the imbalance in the dataset, where exploration was more present than integration, resolution, and non-cognitive presence (exploration > integration > resolution > non-cognitive presence). Balancing imbalanced datasets is a well-known method in machine learning model deployment (Batista et al., 2004).

In sampling scenarios 3 and 4, we built our machine learning classifier after denoising the dataset. In scenario 3, we used only LIWC features, and in scenario 4, we used LIWC features with the other learning traces. A similar method of denoising the dataset was performed by Barbosa et al. (2020). The neural network in both scenarios then achieved the highest performance. Compared to scenarios 1 and 2, this resulted in significantly higher accuracy values (p < 0.01) using a Mann–Whitney U test.

Overall, all four classifiers showed a substantial or almost perfect agreement using a neural-network-based approach. Interestingly, the classifier of the third and fourth scenarios outperformed other classifiers in terms of accuracy using a SMOTEENN sampling approach. Nevertheless, the third and the fourth classifier may not be applicable since it seems impossible to apply over- and undersampling methods to real-life data in real-time.

As already discussed in the introduction, earlier work on automatic classifiers for cognitive presence was mostly based on only one dataset (a dataset of a specific postgraduate software engineering course). Our work now provides a new, fully coded German dataset for future research on cognitive presence in the German language. It is available from the authors upon request.

Summarizing, we found that the automatic classification of cognitive presence based on German-language student posts using a multilayer perceptron is possible with sufficient accuracy. The accuracy we achieved is comparable to similar studies with English-language posts. We achieved the best results using linguistic analysis (LIWC). The other learning traces, which are also hard to extract automatically from the learning management system, are not as crucial for classification as we expected. The essential LIWC features were students’ use of conjunctions and the number of words bigger than six letters, which is consistent with earlier authors (Neto et al., 2018). The classifier seems to classify better if more complex words and sentences are in the students’ written artifacts.

The modest sample size of the number of students in our study may be seen as a limitation. However, we coded over 1,100 posts (with more than 1,500 meaningful units) which is not so different from related work in this area. For instance, Rolim et al., Gašević et al., Kovanović et al., and Joksimović et al. worked with an identical dataset consisting of 1,747 student posts (Gašević et al., 2015; Joksimović et al., 2014; Kovanović et al., 2015; Rolim et al., 2019) .

Our group of students represents a large diversity of adult students coming from varying age groups, countries, and professional backgrounds. We thus feel that our results are of interest to similar settings of postgraduate adult learners in an academic environment.

We used a structured coding process to reach a high level of consensus in the coding that was identical to the coding process of earlier CoI researchers, e.g., Neto et al. (2018). The chosen approach is called “negotiated coding” (Garrison et al., 2006). Negotiated coding starts with a joint training session (in our case, 50 posts) and continues with block-wise coding (in our case, blocks of 150 posts). After each block, the coding is reviewed and negotiated. Interrater agreement before this negotiation is typically low, even with experienced coders. Garrison, for example, achieved interrater agreement of 27–68% per block before negotiation (Garrison et al., 2006). After negotiation, high agreement levels are typically reached (e.g., 98% in Rolim et al., 2019). In our case, we reached 98% consensus, which was an excellent basis for the machine-learning process.

We added learning traces (i.e., attributes of students’ posts that are not purely textual) to our automatic classifier. This idea had to our knowledge not been pursued by other researchers before. Our idea was that these learning traces might provide additional information on the level of cognitive presence. Our results showed that these learning traces did not improve classification accuracy. These learning traces are also not easy to extract automatically from the learning management system. Nevertheless, learning processes may be visible not only in students’ written posts but also in other learning artifacts, which warrants further research in this area.

Further research is necessary to apply our classifier as part of a teacher dashboard. First, we must verify our classifier in further German-language online postgraduate courses in related fields (e.g., medical informatics, computer science, natural sciences) to assess its generalizability in these settings. As the words used by the LIWC tool are not content-specific, it should be possible to use them in other thematic fields or other academic settings. Second, we need to automate the machine learning pipeline from the learning management system and consider aggregating the information for a teacher dashboard. We must address that our classifier can only predict one phase of cognitive presence per post. We thus have to solve the challenge of posts comprising several phases. For a teacher dashboard, we also need to consider the explainability of the presentation to the teacher, since learning analytics applications should build on trust and transparency. A teacher dashboard should thus also allow additional qualitative information on the content of the discussion. Here, tools to summarize written text could be used in addition to our automatic classifier (Rodríguez et al., 2022).

Third, presenting information on cognitive presence may not only be beneficial for the teacher but may also support students in self-regulating their learning. Research has shown that providing cognitive diagrams to students impacts discussion behaviors (Kwon & Park, 2017). Research has also shown that providing cognitive presence information to students may increase the level of cognitive presence (Alwafi, 2022). Our classifier may thus also be considered in future research on a student dashboard. Finally, if we succeed in building an accurate teacher dashboard, research has to evaluate whether the information on the level of cognitive presence has an impact on the activities of the teacher (e.g., fostering discussions) and whether this, in turn, has a measurable impact on the cognitive presence of the students and ultimately on the learning outcome. This may require controlled studies to be designed and carried out.

## Conclusion

We deployed a first German-language classifier for cognitive presence. We see this work as a first step toward a real-time CoI teacher dashboard that could help teachers to monitor their students, especially in a large group. Future research needs to solve the technical and methodological challenges of real-time analysis of students’ posts, such as automatization of the whole pipeline, and evaluate the impact of presenting the information related to cognitive presence to teachers and students.

## Data Availability

The coding guideline and the datasets used and analyzed during the current study are available from the corresponding author upon reasonable request.
